# Surveillance of congenital Zika syndrome in England and Wales: methods and results of laboratory, obstetric and paediatric surveillance

**DOI:** 10.1017/S0950268819001535

**Published:** 2019-09-04

**Authors:** C. Oeser, E. Aarons, P.T. Heath, K. Johnson, A. Khalil, M Knight, R. M. Lynn, D. Morgan, R. Pebody

**Affiliations:** 1 National Infection Service, Public Health England, London, UK; 2 Rare and Imported Pathogens Laboratory, Public Health England, Porton, Salisbury, UK; 3 Institute of Infection and Immunity, St George's, University of London, London, UK; 4 Neonatal Service, Leeds Teaching Hospitals NHS Trust, Leeds, UK; 5 Obstetrics and Maternal-Fetal Medicine, St George's, University of London, London, UK; 6 National Perinatal Epidemiology Unit, University of Oxford, Oxford, UK

**Keywords:** Arboviruses, Congenital (intrauterine) infection, Infectious disease control, Surveillance, Surveillance system

## Abstract

The spread of the Zika virus (ZIKV) in the Americas led to large outbreaks across the region and most of the Southern hemisphere. Of greatest concern were complications following acute infection during pregnancy. At the beginning of the outbreak, the risk to unborn babies and their clinical presentation was unclear. This report describes the methods and results of the UK surveillance response to assess the risk of ZIKV to children born to returning travellers. Established surveillance systems operating within the UK – the paediatric and obstetric surveillance units for rare diseases, and national laboratory monitoring – enabled rapid assessment of this emerging public health threat. A combined total of 11 women experiencing adverse pregnancy outcomes after possible ZIKV exposure were reported by the three surveillance systems; five miscarriages, two intrauterine deaths and four children with clinical presentations potentially associated with ZIKV infection. Sixteen women were diagnosed with ZIKV during pregnancy in the UK. Amongst the offspring of these women, there was unequivocal laboratory evidence of infection in only one child. In the UK, the number and risk of congenital ZIKV infection for travellers returning from ZIKV-affected countries is very small.

## Introduction

In February 2015, health authorities of Caxias, a municipality in the North eastern state of Maranhao in Brazil, reported an outbreak of an unidentified febrile illness characterised by a rash and joint pain, affecting hundreds of people. Viral infections common in this area such as Chikungunya virus were quickly excluded; however, it took several months for the pathogen to be identified. Almost 7000 cases had been notified when an association with Zika virus (ZIKV) was confirmed in May 2015 and the Ministry of Health reported autochthonous transmission of ZIKV for the first time in Brazil [1].

In October 2015, approximately 9 months after the occurrence of the first cases of ZIKV infection, an unusual increase in the number of children born with microcephaly were noted in the city of Recife in Brazil's Northeastern state of Pernumbuco. Similar observations were reported in the neighbouring states of Paraíba and Rio Grande do Norte. Brain scans of affected infants, where available, showed an unusual pattern of pathologies, suggesting a new, thus far not recognised causal agent. An association with ZIKV infection during pregnancy was hypothesised and the Brazilian Government declared a national public health emergency on 11 November 2015. The Pan American Health Organization/World Health Organization (PAHO/WHO) responded by issuing an epidemiological alert; WHO Member States were asked to report increases of congenital microcephaly and other central nervous system malformations through the International Health Regulations (IHR) (https://www.who.int/emergencies/zika-virus/history/en/).

Increasing evidence of an association of ZIKV infection with microcephaly and other neurological disorders was emerging rapidly, and on 1 February 2016, the WHO declared a Public Health Emergency of International Concern. Subsequently, in March 2016, the WHO updated its travel recommendations, advising pregnant women not to travel to areas with ongoing ZIKV outbreaks. Based on the review of the existing literature, in April 2016, a scientific consensus was reached that ZIKV infection during pregnancy is a cause of congenital brain abnormalities, including microcephaly (also referred to as congenital Zika syndrome, CZS). By mid-2016, the virus had spread to many other countries in South and Central America and the Caribbean (https://www.who.int/emergencies/zika-virus/history/en/).

Whilst the mosquito carrying the virus is not established within the UK, every year a large number of people, including pregnant women, visit countries with active ZIKV transmission. In 2015 and 2016, around 3.5 million UK residents travelled to countries affected by ZIKV (https://www.ons.gov.uk/peoplepopulationandcommunity/leisureandtourism/datasets/overseastravelandtourism); it was estimated that approximately 28% of those were women of childbearing age. Travel advice for pregnant women or those planning pregnancy was issued by Public Health England (PHE) in January 2016; however, there was no noticeable decrease in travel activity to affected countries in 2016 (https://www.ons.gov.uk/peoplepopulationandcommunity/leisureandtourism/datasets/overseastravelandtourism).

At the early stage of the epidemic, little was known about the clinical presentation or the level of risk that ZIKV infection posed to pregnant women travelling to affected areas. In this rapidly evolving situation, only limited evidence on the range of presentations of CZS was available, often making it necessary to base guidance and recommendations on single case reports.

In light of this uncertainty and the rapid spread of the virus, surveillance of newborn babies who were potentially exposed to ZIKV infection during pregnancy needed to be established urgently. The aim of the surveillance was to document numbers of potential cases; to estimate the risk to travellers returning to the UK from countries where there is ongoing ZIKV transmission, as well as, if required, aid planning for future services of care.

Several systems exist within the UK to facilitate a rapid response to public health emergencies and surveillance of rare congenital infections; the British Paediatric Surveillance Unit (BPSU), the UK Obstetric Surveillance System (UKOSS) and the PHE Rare and Imported Pathogens Laboratory (RIPL).

By deploying and adapting these existing systems, and combining their results, we aimed to establish the number of pregnant women potentially exposed, identify affected children, estimate the risk to the travelling UK population and to help further knowledge on the natural history of CZS.

This article describes the methods and findings of surveillance conducted by the BPSU, UKOSS and RIPL.

## Methods

### The British Paediatric Surveillance Unit

The BPSU (http://www.rcpch.ac.uk/bpsu), a joint initiative of the Royal College of Paediatrics and Child Health (RCPCH), PHE and the UCL Great Ormond Street Institute of Child Health (ICH), supports surveillance of rare childhood disorders. The unit also aims to enable organisations to undertake a rapid response to emerging public health threats and to facilitate research for improvement in prevention, treatment and planning of services for rare disorders.

A reporting card (‘Orange Card’) with a list of conditions is sent electronically each month to over 3300 consultant paediatricians and other specialists in the UK and also (as the only one of the three surveillance systems) the Republic of Ireland (ROI). Clinicians complete the card, notifying the BPSU of any cases of diseases under surveillance or ‘nothing to report’. Reporting clinicians are asked to complete a questionnaire for cases identified. PHE has approval under Section 251 of the NHS Act 2006 to process confidential patient information for public health purposes.

For surveillance of Zika congenital syndrome, all infants ⩽6 months of age with a head circumference >2 standard deviations below the mean for gestational age and sex (i.e. below the 2nd centile), or any neurological abnormality requiring investigation, whose mother has travelled to a country with active Zika transmission during pregnancy or in the 3 months prior to conception were included. In February 2017, surveillance was extended for a further 12 months until 31 March 2018 and inclusion criteria were extended to include infants ⩽12 months of age.

Countries with active Zika transmission were identified through the European Centre for Disease Prevention and Control (ECDC) which maintained an up-to-date list of countries with current active ZIKV transmission and transmission status 9 months previously (see https://ecdc.europa.eu/en/publications-data/current-zika-transmission-worldwide and https://ecdc.europa.eu/en/publications-data/zika-transmission-past-nine-months).

Due to the limited existing data, and because the evidence on the nature, clinical presentation and laboratory diagnosis of the condition was evolving throughout the course of the outbreak, confirmation of cases and non-cases was anticipated to be challenging. Therefore, an expert panel consisting of virologists, public health experts, paediatric infectious diseases and neonatal physicians was convened to discuss each case when reported.

**Fig. 1.**
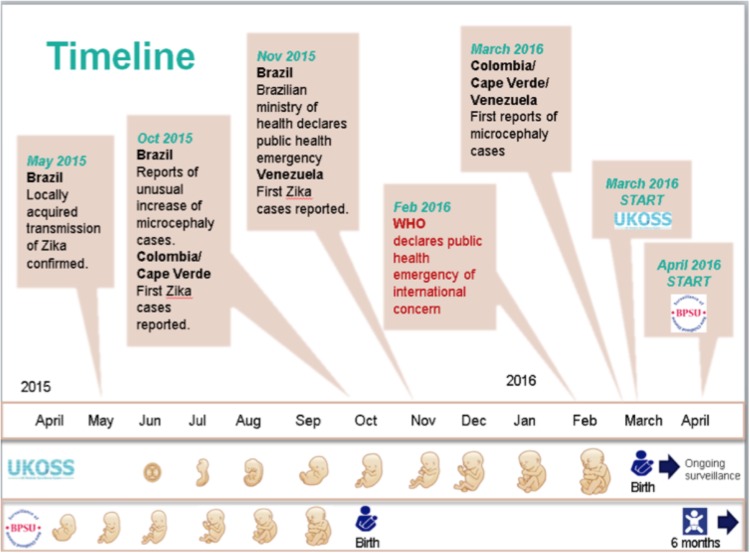
Start of UKOSS and BPSU surveillance in relation to the timeline of the outbreak.

BPSU surveillance commenced on 1 April 2016 and concluded on 31 March 2018. The case definition described above, including children ⩽6 months of age, enabled identification of potential cases born to mothers infected at the end of the first trimester at the start of the outbreak in April 2015 (see Fig. 1).

A follow-up visit for a developmental assessment was performed at 24 months of age. Due to small numbers, detailed descriptions of the cases would make patients identifiable; therefore, results are presented as aggregates.

### The UK Obstetric Surveillance System

UKOSS (https://www.npeu.ox.ac.uk/ukoss), based at the National Perinatal Epidemiology Unit, University of Oxford, investigates uncommon disorders in pregnancy. Similar to the BPSU, this active surveillance system operates as a prospective monthly case-collection scheme. Each UK hospital with a consultant obstetric unit receives a monthly email with a list of conditions under surveillance. Clinicians report via an electronic link any cases that occurred in the previous month, or if none, submit a nil return. For a case, detailed information is sought through a data collection form [2]. This methodology has been approved by the North London Multi-centre Research Ethics Committee R1 (ref10/H0717/20). Unlike the BPSU, this surveillance did not cover ROI.

Surveillance of CZS aimed to assess the risk of having an adverse pregnancy outcome after travelling to a country with active ZIKV transmission. For this purpose, clinicians were asked to report two different groups of women:

1. Any pregnant woman with a history of travel to a country with active ZIKV transmission during pregnancy or in the 4 weeks before conception and no adverse pregnancy outcome.
2. Any pregnant woman with a history of travel to a country with active ZIKV transmission during pregnancy or in the 4 weeks before conception with an adverse pregnancy outcome.

Countries with active Zika transmission were identified as above.

Thus, numbers of all pregnant women with a relevant travel history at an antenatal appointment were reported to UKOSS; subsequently, detailed information was sought from cases if an adverse pregnancy outcome was identified.

UKOSS surveillance commenced on 1 March 2016 and was completed by 28 February 2017. The case definition enabled inclusion of potential infections occurring at the beginning of the outbreak, including pre-conception (see Fig. 1). In combination with surveillance conducted by the BPSU, these case definitions aimed to enable identification of cases over the entirety of the outbreak.

### The Rare and Imported Pathogens Laboratory

RIPL is a PHE specialist centre for advice and diagnosis for unusual and travel-associated viral and bacterial infections in the UK. It provides medical and laboratory specialist services to the NHS and other healthcare providers and is the sole provider for NHS testing for ZIKV. Both molecular and serology tests are used. (https://www.gov.uk/guidance/zika-virus-sample-testing-advice). ZIKV testing in the ROI is not provided by RIPL.

Testing for ZIKV is provided for any patient who has or has had a rash illness or other symptoms suggestive of ZIKV infection that began whilst in any country with active ZIKV transmission, or within 2 weeks of leaving that country. Testing is also provided for patients presenting with typical Zika-like symptoms whose male sexual partner had travelled within the last 3 months from a country with active ZIKV transmission.

As described by Petridou *et al*. [3], PCR testing has been performed on suspected symptomatic cases since November 2015, and serological testing was introduced at a later stage, with antibody testing performed retrospectively on all earlier samples received from a symptomatic pregnant woman. Testing of symptomatic returning travellers or sexual contacts for ZIKV is ongoing.

Detection of ZIKV RNA in any sample is considered diagnostic of infection. Detection of ZIKV IgM and/or IgG is interpreted as described previously [3].

## Results

*BPSU*: From 1 April 2016 to 31 March 2018, eight suspected cases were reported from the UK (7) and ROI (1). Of those, one case was excluded due to not fulfilling the inclusion criteria and three were excluded as laboratory tests on the mother and baby were reported as negative for ZIKV. The remaining four possible cases were followed up until 27 months of age.

Maternal travel destinations included countries in South America, the Caribbean and Asia.

No diagnostic ZIKV testing was performed for some of the investigated possible cases as recruitment at the start of the epidemic was retrospective, including infants up to the age of 6 months whose mothers had had no symptoms suggestive of ZIKV infection. Consequently, it is possible that the untested children did not in fact have congenital ZIKV infection. One of the infants had laboratory-proven ZIKV infection reported by RIPL (see below). The four infants presented with a range of clinical features including prematurity, small for gestational age, micro- and macrocephaly, facial abnormalities and craniofacial disproportion, intracranial abnormalities (calcifications, cortical/subcortical atrophy, ventricular dilation and dysmorphia, subependymal cysts, corona radiata changes, grey matter heterotopia), nose abnormalities, retinal abnormalities and visual impairment, tremors, hyperreflexia, cerebral palsy and swallowing difficulties.

Follow-up occurred until 27 months of age and information on developmental progress was received on all four cases. Findings included reports of normal development (1/4) as well as seizures, delayed language, neuromotor and social skills (3/4), visual (1/4) and hearing impairment (1/4). Other features noted were microcephaly and dysmorphic features (2/4).

*UKOSS*: From 1 March 2016 to 28 February 2017, 827 women were reported to have travelled from the UK to countries with active Zika transmission during pregnancy or in the 4 weeks prior to conception. Of these, 10 were reported to have adverse outcomes. Data collection forms were received from nine of these cases. Three of those had symptoms suggestive of ZIKV infection, four were tested and reported negative for ZIKV. Of the remaining five (0.6%), three had a miscarriage (3/5, two at 10 weeks of gestational age, for one gestational age was unknown) and two an intrauterine death (2/5 at 27 and 40 weeks of gestational age). Travel destinations were South and Central America and the Caribbean for four cases, and only one case had travelled to Asia.

*RIPL*: Detailed results of RIPL surveillance over the 2-year period 2016–2017 are described elsewhere [3]. In summary, 16 (1%) of the 1256 pregnant women tested for ZIKV infection were diagnosed with either PCR-confirmed infection, seroconversion, ‘probable’ or ‘likely’ infection. Of these, two had a miscarriage and 14 proceeded to give birth; in one of those, *in utero* ZIKV infection was subsequently confirmed.

There were no reports of adverse pregnancy outcomes through the presumed sexual acquisition of ZIKV infection, and indeed RIPL identified only a single instance of sexually acquired infection in the UK [3].

**Fig. 2.**
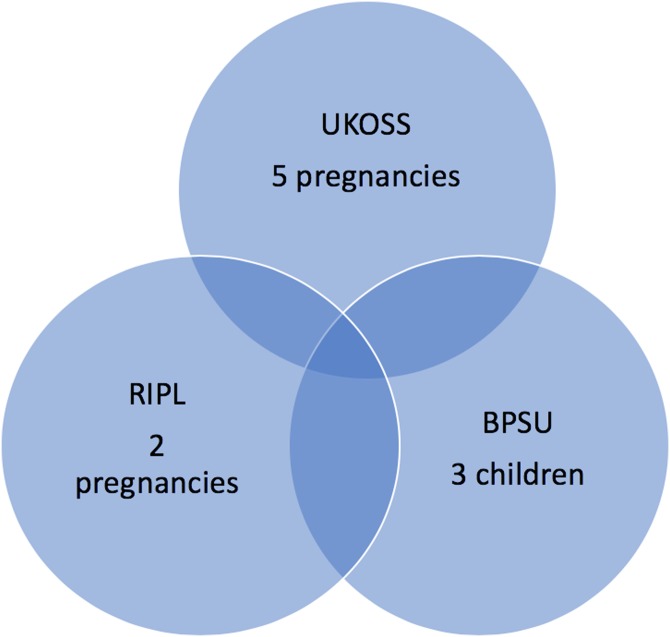
Numbers of adverse pregnancy outcomes potentially associated with ZIKV infection as reported by the three surveillance systems (note UKOSS surveillance was conducted over 1 year, RIPL and BPSU over 2 years).

In summary, among 827 pregnant women in the UK, reported to have been potentially exposed to ZIKV over 1 year, 0.6% experienced an adverse pregnancy outcome. One per cent of 1256 potentially exposed symptomatic pregnant women tested in the UK over 2 years were diagnosed with ZIKV infection and one out of the subsequent 14 live births exposed to ZIKV *in utero* became infected. Three infants with abnormalities potentially attributable to congenital ZIKV infection were identified by BPSU during 24 months of surveillance. One child with laboratory-proven ZIKV infection was reported by RIPL as well as BPSU (Fig. 2).

## Discussion

A large proportion of countries in the Southern hemisphere have reported past or current ZIKV transmission since the start of the outbreak, including countries or areas in South East Asia and Africa and the USA. However, since the peak of the epidemic in 2016, reported ZIKV infections have continuously decreased in the majority of countries, and in some, virus transmission has been interrupted (https://ecdc.europa.eu/sites/portal/files/documents/zika-risk-assessment-9-april-2019.pdf).

Despite many women visiting countries with active Zika transmission during pregnancy, only a small number of adverse outcomes potentially associated with ZIKV infection during pregnancy have been reported from the various surveillance systems. This indicates that, even during the peak of the ZIKV epidemic, the risk to the UK travelling population was very low.

All cases reported to the BPSU were evaluated by an expert group comprised of members of different medical specialties including virology, neonatology and paediatric infectious diseases; however, a definitive cause for the presenting abnormalities could not be determined for some. This is due to a lack of evidence on the range of presentations of CZS as well as the fact that serological testing was not performed for those cases identified retrospectively at the very beginning of the outbreak and whose mothers had not had any symptoms suggestive of ZIKV infection. Moreover, causes for microcephaly and accompanying neurological abnormities are extensive and include genetic and metabolic conditions as well as infections and teratogenic factors. In approximately 40% of cases with congenital microcephaly, the aetiology is unknown [4].

Prompt ZIKV testing and detailed follow-up, including standardised neurodevelopmental assessments, together with increasing evidence from reports and case studies describing the progress of children with CZS from countries affected by the outbreak will guide the further evaluation of possible cases in the future.

Employing two case definitions in the UKOSS surveillance facilitated active reporting of adverse pregnancy outcomes and provided an estimate of the number of women who had travelled to affected countries during pregnancy. However, it needs to be taken into consideration that this number (*n* = 827) is likely to be an underestimate. Based on the provided estimates of numbers of women of child-bearing age travelling to affected countries and a general fertility rate of ~60/1000 (https://www.ons.gov.uk/peoplepopulationandcommunity/birthsdeathsandmarriages/livebirths/bulletins/birthsummarytablesenglandandwales/previousReleases), when assuming an average duration of stay of approximately 2 weeks, an estimated 1225 pregnant women would have been potentially exposed to ZIKV over 1 year, indicating the above calculated risk of 0.6% may be lower.

It was not possible to attribute specific adverse outcomes to ZIKV infection during pregnancy for these cases identified by UKOSS, possibly as many of the adverse outcomes occurred early on in pregnancy, before testing would have been considered. However, through this method, we were able to establish that the risk of adverse pregnancy outcomes in pregnant women travelling to countries affected by ZIKV is low and does not exceed the expected background rate of either of the adverse pregnancy outcomes at observed gestational ages (11–22% for miscarriages [5] and 0.4% for intrauterine foetal death [6]).

Testing for ZIKV performed at RIPL over the 2-year period 2016–2017 showed that the number of apparently symptomatic women testing positive after potential exposure to ZIKV through travel is also low (1%). This highlights that, even at the time of the epidemic, the likelihood of a pregnant traveller acquiring ZIKV infection was very small. Only one of 14 children born to women diagnosed with ZIKV also acquired the infection.

The presence of established systems provided a platform to enable the timely organisation of surveillance of CZS in returning travellers in the UK. Laboratory surveillance was available from early on in the outbreak, UKOSS surveillance was established within 1 month and BPSU surveillance within 2 months of the WHO declaration of the Public Health Emergency of International Concern. The variation and minimal overlap of the results of these surveillance systems demonstrate the importance of considering different approaches of surveillance, spanning the entire time period relevant to the outbreak and encompassing several disciplines with distinct case definitions as employed here, particularly when faced with the challenges of a previously unknown infection.

Combining results of the different surveillance systems confirmed that the risk of adverse pregnancy outcomes because of ZIKV infection overall is low. Employing established surveillance systems at an early stage of the outbreak facilitated information on the risk to the travelling UK population. The prompt and effective response of these separate surveillance systems provided reassurance that suitable approaches are in place to respond to similar threats in a coordinated effort in the future.

International Networks such as the International Network of Paediatric Surveillance Units (INOPSU) and International Network of Obstetric Survey Systems (INOSS) of those countries with similar paediatric and obstetric surveillance systems exist and could be used for rapid international studies of congenital infections in the future.
